# The transformative power of transformers in protein structure prediction

**DOI:** 10.1073/pnas.2303499120

**Published:** 2023-07-31

**Authors:** Bernard Moussad, Rahmatullah Roche, Debswapna Bhattacharya

**Affiliations:** ^a^Department of Computer Science, Virginia Tech, Blacksburg, VA 24061

**Keywords:** protein structure prediction, neural networks, transformers, deep learning

## Abstract

Transformer neural networks have revolutionized structural biology with the ability to predict protein structures at unprecedented high accuracy. Here, we report the predictive modeling performance of the state-of-the-art protein structure prediction methods built on transformers for 69 protein targets from the recently concluded 15th Critical Assessment of Structure Prediction (CASP15) challenge. Our study shows the power of transformers in protein structure modeling and highlights future areas of improvement.

In 2020, DeepMind’s AlphaFold2 method (1) made a major scientific breakthrough in predicting the three-dimensional (3D) structure of proteins from the amino acid sequences. AlphaFold2 demonstrated remarkable performance in the 14th edition of the biennial Critical Assessment of Structure Prediction (CASP14) meeting, attaining better predictive modeling accuracy than ever before (2). Notably, DeepMind released the source code of AlphaFold2 with an open-source license along with an extensive database of predicted protein structures (3). Meanwhile, the scientific community has been investigating ways to explore, exploit, and extend AlphaFold2 to further improve the state of the art of protein structure prediction for advancing structural biology research (4–6).

The core of the AlphaFold2 framework consists of transformer neural networks powered by the attention mechanism (7), a sequence transduction model originally proposed to draw global dependencies between input and output. What makes the transformer architecture particularly powerful is its ability to model long-range relationships in the input sequence beyond their sequential neighborhoods. AlphaFold2 harnessed the power of transformers by formulating a “two-track” neural architecture, named Evoformer, to jointly embed the evolutionary and spatial relationships between the amino acids using multiple sequence alignment (MSA) and pairwise representations, followed by a structure module based on equivariant transformer architecture with invariant point attention. RoseTTAFold (5) extended the two-track architecture of AlphaFold2 by adding a third track operating in 3D coordinate space using SE (3)-transformer (8). The key principle of the three-track RoseTTAFold network is to process MSA, pairwise, and coordinate information simultaneously, with accuracies approaching those of AlphaFold2.

It is worth noting that both AlphaFold2 and RoseTTAFold rely on the availability of evolutionary information in the form of MSAs, which are not always as abundant as these methods demand, such as with orphan proteins or rapidly evolving proteins. Protein language models (PLMs) aim to overcome this limitation by pretraining a family of transformer-based models of protein sequences. For example, OmegaFold (4) introduced a transformer-based PLM called OmegaPLM to learn single- and pairwise-residue embeddings as features that are fed into a geometry-inspired transformer block named Geoformer. ESMFold (6) trained a massive PLM called ESM-2 based on an encoder-only transformer architecture (7) and leverages the evolutionary information captured by the model using a modified Evoformer block of AlphaFold2 to remove its dependence on MSAs. Unlike AlphaFold2 or RoseTTAFold, neither OmegaFold nor ESMFold rely on MSAs or use known structures as templates.

Despite their methodological differences, the state-of-the-art methods for protein structure prediction have two things in common: 1) Their underlying neural network architectures use transformers as building blocks, and 2) their source codes are freely available with open-source license. That is, an open-source ecosystem of transformer-based neural networks is having a transformative effect in the field of protein structure prediction.

Given these recent advances, a natural question arises: how far have we progressed in predicting protein structures since the breakthrough of AlphaFold2 in 2020? It is widely accepted by the structure prediction community that blind structure prediction tests are needed to objectively assess the performance of new protein structure prediction methods. Fortunately, the recently concluded CASP15 meeting provides an excellent testbed to evaluate these emerging methods over the same set of proteins. Here, we benchmark the predictive modeling performance of AlphaFold2, RoseTTAFold, ESMFold, and OmegaFold on a set of 69 CASP15 single-chain protein targets with available experimental coordinates. Our benchmarking differs from the CASP15 official assessment in multiple ways. First, we downloaded the open-source software implementations for all methods and employed the modeling pipeline in fully automated mode with default parameter settings, without any manual interventions. Second, we evaluated single-chain predictive modeling accuracies by directly comparing the full-length predictions against the experimental coordinates, without splitting them into domains. Additional domain-level analyses were performed on multidomain proteins to examine the relative accuracies of individual domains and their packing. Third, we used static databases, libraries, and model weights released before CASP15, without intermediate updates. Our study highlights the similarities and differences among contemporary methods, reveals the challenges, and emphasizes future areas of improvement.

## Results

**Fig. 1.**
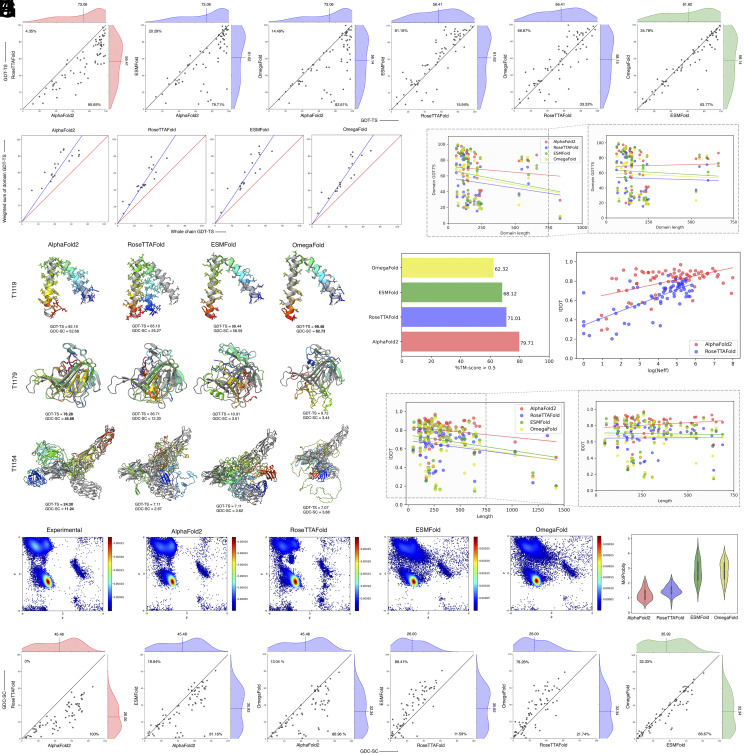
Performance benchmarking of the state-of-the-art protein structure prediction methods on the CASP15 dataset. (*A*) GDT-TS score comparisons. The dashed lines represent the mean performance with the percentages reported in the *Top* *Left* indicating that the method in the *y* axis outperforms the method in the *x* axis, and vice versa for the *Bottom* *Right*. (*B*) Grishin plot analysis. (*C*) Domain-level GDT-TS scores against the length of the domains with the inset showing the domains having lengths less than 750 residues. The lines represent linear fit to the data. (*D*) Three representative CASP15 targets with the predictions (in rainbow) superimposed on the experimental structures (in gray). Bold numbers indicate the best performance. (*E*) Correct overall topology prediction performance in terms of %TM-score >0.5. (*F*) lDDT scores of MSA-based methods against the MSA depth measured by the logarithm of Neff. The lines represent linear fit to the data. (*G*) lDDT scores against the length of the protein targets with the inset showing the targets having lengths less than 750 residues. The lines represent linear fit to the data. (*H*) Ramachandran plot analysis. The color code ramps from blue to red for low to high density. (*I*) MolProbity score distributions. (*J*) GDC-SC score comparisons similar to *A*.

Fig. 1*A* shows the backbone accuracies of various methods and their head-to-head comparisons using the global distance test (GDT-TS) metric (9). AlphaFold2 attains the best performance with the highest mean GDT-TS score of 73.06, convincingly outperforming all other methods. ESMFold attains the second-best performance in terms of backbone positioning, attaining a mean GDT-TS score of 61.62. It is interesting to note that PLM-based ESMFold outperformed MSA-based RoseTTAFold for more than 80% of the cases and attained a higher mean GDT-TS score. In contrast, AlphaFold2 outperforms ESMFold for nearly 80% of the cases. Among the two PLM-based methods, ESMFold attained better overall accuracy in terms of backbone positioning compared to OmegaFold.

For the 19 multidomain proteins according to the official CASP15 domain definitions, we examined the relative accuracies of 45 individual domains and their packing by inspecting the Grishin plots (10), which are correlation plots between the weighted sum of domain (*y* axis) and the whole chain GDT-TS (*x* axis). The Grishin plots shown in Fig. 1*B* suggest that the individual domains are predicted reasonably well, whereas their packing is not. The GDT-TS scores of the 45 individual domains themselves are largely size independent (Fig. 1*C*), particularly when the few domains with length more than 750 are discarded (Fig. 1 *C*, *Inset*). Collectively, the results indicate that the source of the error in large multidomain proteins is often due to the misprediction of domain orientations even when the individual domains are accurately predicted. Clearly, there is room for improvement in the relative packing of individual domains.

Three representative CASP15 proteins are shown in Fig. 1*D*. For the small helical target T1119, all methods except RoseTTAFold attained a GDT-TS score of more than 90. Two PLM-based methods performed the best with OmegaFold attaining a near-perfect GDT-TS score of 99.48, closely followed by ESMFold (GDT-TS score of 98.44), outperforming the other methods including AlphaFold2. In contrast, for the target T1179 having a nontrivial topology, AlphaFold2 attained the highest GDT-TS score of 78.28, much better than RoseTTAFold (GDT-TS score of 36.71), whereas the PLM-based methods essentially generated random predictions (GDT-TS score < 11). Finally, for the large multidomain target T1154 with a complex topology, all methods performed poorly; even the top-performing AlphaFold2 attained a low GDT-TS score of 24.3. That is, despite yielding near-perfect predictive accuracy for small proteins with simple topology, state-of-the-art methods are still far from perfect for medium to large proteins with complex topology.

In terms of correct overall topology prediction, quantified by the percentage of template modeling score (TM-score) > 0.5 (11), all methods performed quite well (Fig. 1*E*) with AlphaFold2 attaining the highest performance close to 80%. RoseTTAFold attained the second-best performance with just over 70%. That is, MSA-based methods attained better correct overall topology prediction performance compared to PLM-based approaches. We investigated whether the accuracy of MSA-based methods is related to the availability of evolutionary information by analyzing the MSA depth measured by the logarithm of the normalized number of effective sequences (Neff) against the local-distance difference test (lDDT) metric (12). The results show (Fig. 1*F*) that both AlphaFold2 and RoseTTAFold are somewhat MSA dependent, with RoseTTAFold exhibiting more MSA dependence. We also examined the relationship between protein size and lDDT metric for all four methods. Despite the apparent trend of a decline in prediction accuracy as the protein size increases (Fig. 1*G*), the accuracy is in fact largely size independent when the few targets with length more than 750 are discarded (Fig. 1 *G*, *Inset*).

How does the stereochemistry of the predicted structural models compare with the experimental structures? The Ramachandran plots (13), capturing the ϕ, ψ backbone torsion angle distributions (Fig. 1*H*), and the MolProbity score (14) distributions (Fig. 1*I*), quantifying the estimated deviations of the predicted structural models from their ideal crystallographic resolutions, reveal that the stereochemistry of the MSA-based methods AlphaFold2 and RoseTTAFold are closer to experimental observations than that of the PLM-based methods ESMFold and OmegaFold. The PLM-based methods in particular exhibit noticeably lower stereochemical qualities having physically unrealistic local structural regions, limiting their applicability to calculations based on the predicted whole chain coordinates such as electrostatic potential, accessibility, etc.

With respect to the side-chain positioning, measured by the global distance calculation for side-chains (GDC-SC) metric (15), there is considerable room for improvement (Fig. 1*J*). While the most accurate method is still AlphaFold2, which outperformed the other methods for more than 80% of the cases, the mean GDC-SC score of AlphaFold2 fell short of 50. It is interesting to note that PLM-based methods ESMFold and OmegaFold outperformed MSA-based RoseTTAFold for a large majority of cases and attained higher mean GDC-SC scores. The case study presented in Fig. 1*D* also underscores the low GDC-SC scores for almost all targets. Improved side-chain packing algorithms will undoubtedly benefit MSA- and PLM-based methods alike.

## Discussion

Despite the dramatic progress in protein structure prediction with transformer-based neural networks trailblazed by AlphaFold2, our study reveals two lingering issues. First, accurate prediction of large multidomain proteins with complex topology remains challenging. For some of such targets, none of the methods were able to predict even the correct overall topology with appropriate domain orientations. Adjusting the transformer model architecture by emulating the principles of interprotein interactions for modeling intraprotein domain–domain interactions may be necessary for large multidomain proteins. Second, the accuracy of side-chain positioning remains low for all methods. Structure prediction algorithms will benefit from accurate side-chain packing, possibly by incorporating side-chain optimization in the neural architecture.

The rise of PLMs clearly offers some unique advantages by being applicable even for proteins that do not have identifiable homologous sequences in the current sequence databases and bypasses the computational overhead of MSA searching. However, the performance of ESMFold and OmegaFold in our benchmarking raises the question: have PLM-based methods already eliminated the need for evolutionary information? Probably not yet. But PLM can be effective for small proteins with simple topology. Combining the strengths of MSA and PLM may be a promising avenue for further progress, broadening the applicability of predictive protein modeling in structural biology.

## Materials and Methods

We considered 69 single-chain protein targets from CASP15 having length less than 3,000 residues with available experimental coordinates. We obtained the sequences of these target proteins from the CASP15 website and predicted their structures using publicly-available versions of AlphaFold2, RoseTTAFold, OmegaFold, and ESMFold (*SI Appendix*) and subsequently evaluated the predictive modeling performance using standard evaluation metrics including GDT-TS (9), TM-score (11), lDDT (12), MolProbity (14), and GDC-SC (15) (*SI Appendix*).

## Supplementary Material

Appendix 01 (PDF)

Dataset S01 (GZ)

## Data Availability

Predicted protein structural models in Protein Data Bank (PDB) format and metrics calculated to benchmark the predictive modeling performance have been deposited in https://github.com/Bhattacharya-Lab/CASP15 (10.5281/zenodo.7682977) (16). All other data are included in the article and/or supporting information.
