# Supplementary material for: The transformative power of transformers in protein structure prediction
Source: Proc Natl Acad Sci U S A. 2023 Jul 31;120(32):e2303499120. doi: 10.1073/pnas.2303499120 (PMC10410766; doi:10.1073/pnas.2303499120)
Supplement: Supplementary file 1 — Appendix 01 (PDF) [file pnas.2303499120.sapp.pdf]

PNAS

Supporting Information for
The transformative power of transformers in protein structure prediction.

Bernard Moussad[a,1], Rahmatullah Roche[a,1], Debswapna Bhattacharya[a, *]

[a]Department of Computer Science, Virginia Tech, Blacksburg, VA 24061, United States of America.

* Debswapna Bhattacharya

Email: dbhattacharya@vt.edu

This PDF file includes:

Supporting text

Supporting Information Text

Installation and runtime parameter settings of the protein structure prediction methods

We used four state-of-the-art protein structure prediction methods built on transformers for predicting structural models for 69 CASP15 single-chain protein targets. All four methods were installed in our in-house 64-bit Linux cluster powered by a 48-GB NVIDIA A40 GPU. Below, we describe the installation and runtime parameter settings of the structure prediction methods.

AlphaFold2. AlphaFold2 (1) was installed via a docker container from https://github.com/deepmind/alphafold downloaded on August 24, 2022. The maximum template date selected was May 05, 2022 in order to not conflict with the start date of CASP15. The full set of genetic databases was utilized by setting the db_preset parameter to 'full_dbs'. We used the default 3 rounds of recycling followed by the relaxation step to improve local geometry. The top-ranked predicted structural model was selected having the highest predicted LDDT (pLDDT) score using the self-assessment module of AlphaFold2.

RoseTTAFold. RoseTTAFold (2) was installed via an Anaconda virtual environment from https://github.com/RosettaCommons/RoseTTAFold downloaded on August 25, 2022. The databases required in RoseTTAFold were released prior to the start of CASP15. For inference, the pyRosetta-based version was used instead of the end-to-end version as the latter exhibited poorer performance compared to the former according to the results reported in the published article of RoseTTAFold (2). The top-ranked predicted structural model was selected using DeepAccNet (3) accuracy estimation.

ESMFold. ESMFold (4) was installed via an Anaconda virtual environment from https://github.com/facebookresearch/esm downloaded on November 2, 2022. Being a method based on protein language model (pLM), ESMFold is free from any database dependencies. The pretrained ESM-2 pLM with 15 billion parameters was used. We used the default 4 rounds of recycling, generating a single prediction for each target that was used as the predicted structural model.

OmegaFold. OmegaFold (5) was installed into a Python virtual environment via the pip installable repository from https://github.com/HeliXonProtein/OmegaFold downloaded on September 6, 2022. The pretrained pLM called OmegaPLM with 670 million parameters was used. Similar to ESMFold, OmegaFold is free from any database dependencies. We used the default 10 rounds of recycling, generating a single prediction for each target that was used as the predicted structural model.

Evaluation metrics and performance assessment

To evaluate the accuracy of the protein structural models predicted by AlphaFold2, RoseTTAFold, ESMFold, and OmegaFold, we used a set of widely used metrics, including GDT-TS (6), GDC-SC (7), TM-score (8) and lDDT (9). The experimental structures were obtained from the CASP15 website at https://predictioncenter.org/download_area/CASP15/targets/ to evaluate the accuracy of the predicted structural models. For evaluating the domain-level prediction accuracy, we used the official domain definitions from CASP15 available at: https://predictioncenter.org/casp15/domains_summary.cgi. Below, we provide a brief description of the evaluation metrics and performance assessment.

GDT-TS. Global distance test total score (GDT-TS) (6) measures the global structural similarity between two protein structures. Specifically, the score quantifies the percentage of residues (using the C_α atoms in particular) in a structural model that fall within specific distance thresholds of the corresponding residues in the reference structure, after optimal structural superposition as implemented in the local/global alignment (LGA) program (6). Four distance thresholds of 1Å, 2Å, 4Å, and 8Å are used for GDT-TS calculation as follows:

$$GDT\_TS = \frac{P1 + P2 + P4 + P8}{4} \quad [1]$$

where Pd represents the percentage of residues in the model that are superimposed with the corresponding residues of the reference structure within the distance threshold of dÅ. The GDT-TS score ranges from 0 to 100, with higher scores indicating greater structural similarity between the model and the reference structure.

GDC-SC. Global distance calculation for side-chains (GDC-SC) (7) is a side-chain specific superposition-based global accuracy metric implemented in the LGA program (6) for measuring the accuracy of the side-chain positioning in a protein structural model. GDC-SC uses residue-specific representative side-chain atoms during the comparison with the reference structure considering 10 different distance thresholds ranging from 0.5Å to 5Å with a step size of 0.5Å as follows:

$$GDC\_SC = \frac{100 * 2 * (k * Pa_1 + (k - 1) * Pa_2 \ldots + 1 * Pa_k)}{(k + 1) * k} \quad [2]$$

where k = 10. GDC-SC score ranges from 0 to 100, and a higher score indicates better accuracy of the side-chain positioning.

For each of 69 full-length targets from CASP15, we evaluated the global backbone and side-chain accuracies of the structural models predicted by AlphaFold2, RoseTTAFold, ESMFold, and OmegaFold by comparing the predictions against the experimental structures using the LGA program and calculating the GDT-TS and GDC-SC scores.

TM-score. Template modeling score (TM-score) (8) is a structural alignment-based evaluation metric to calculate the topological similarity of two protein structures, calculated as follows:

$$TM\_score = Max\left[\frac{1}{L_N}\sum_{i=1}^{L_T}\frac{1}{1+\left(\frac{d_i}{d_0}\right)^2}\right] \quad [3]$$

where L_N represents the length of the reference protein, L_T represents the length of the aligned residues in the predicted structural model, d_i is the distance between the ith pair of aligned residues, and d_0 is a scale to normalize the TM-score in a way that the magnitude of the average TM-score for random protein pairs is independent of the size of the proteins. Max denotes the maximum value after optimal structural superposition between the predicted structural model and the reference protein.

Ranging from 0 to 1, TM-score provides a measure of the topological similarity of a protein structural model to a reference structure, with higher scores indicating better similarity. Particularly, TM-score > 0.5 indicates that the predicted structural model has a similar overall topology to the reference structure (10). We calculated the TM-scores of the structural models predicted by AlphaFold2, RoseTTAFold, ESMFold, and OmegaFold for the 69 CASP15 targets by comparing them against the experimental structures. For each method, we then calculated the correct overall topology prediction by identifying the predicted structural models with TM-score > 0.5.

lDDT. The local distance difference test (lDDT) (9) measures the accuracy of a predicted structural model against the reference structure in a superposition-free way by computing the agreements between the local distances (L) of atom-pairs in a structural model within a certain inclusion radius (default 15Å) to that of the reference structure. The local distances are considered 'preserved' if they fall within specific distance thresholds (typically, 0.5Å, 1Å, 2Å, and 4Å), and lDDT calculates the fraction of preserved distances as a measure of the accuracy of the

predicted structural model. The final lDDT score is obtained through averaging the fractions of local distances across all distance thresholds.

We calculated the global lDDT scores to evaluate the structural models predicted by AlphaFold2, RoseTTAFold, ESMFold, and OmegaFold against the experimental structures. The global lDDT score ranges from 0 to 1, with higher scores indicating higher predictive modeling accuracy.

Assessment of multi-domain proteins. To investigate the domain-level predictive modeling accuracy, we analyzed the subset of multi-domain targets out of 69 full-length targets from CASP15, resulting in a total of 45 domains. We measured the domain-level GDT-TS for each predicted structural model with respect to the corresponding 45 experimental domains and subsequently computed the weighted-sum GDT-TS as follows:

$$weighted\ sum\ GDT\_TS = \frac{L1 * GDT\_TS(D1) + L2 * GDT\_TS(D2) + \ldots + Ln * GDT\_TS(Dn)}{L1 + L2 + \ldots + Ln} \ldots\ldots\ldots\ldots [4]$$

where Li is the length of domain i, $GDT\_TS(Di)$ is the GDT-TS score for domain i, and n represents the number of domains in a full-length target. Using all the multi-domain targets, we analyzed the Grishin plots (11) for AlphaFold2, RoseTTAFold, ESMFold, and OmegaFold to investigate how the full-length target GDT-TS compares to the weighted sum GDT-TS.

Ramachandran plot analysis. In addition to measuring the accuracy of the predicted structural models by comparing against the experimental structures, we analyzed the Ramachandran plot (12) which captures the joint distribution of the backbone torsion angle pairs ϕ and ψ. A Ramachandran plot offers a standard local quality check of protein structures often used by crystallographers to detect possible angular outliers since not all values of ϕ and ψ are equally frequent, and many combinations are never observed due to steric constraints. We examined how closely the Ramachandran plots of predictions from AlphaFold2, RoseTTAFold, ESMFold, and OmegaFold matched the Ramachandran plot for the corresponding experimental structures.

MolProbity analysis. To further investigate the quality of the predicted structural models at the atomic detail, we used the MolProbity score (13), which is a composite evaluation metric that analyzes the steric clashes between atoms in a protein structure, the percentages of Ramachandran outliers, side-chain rotamers outliers, and the C_β outliers. The MolProbity score provides a quantitative measure of the estimated deviation of a structural model from its ideal crystallographic resolution, with lower score indicating higher quality. We analyzed the MolProbity scores for predicted structural models from AlphaFold2, RoseTTAFold, ESMFold, and OmegaFold.

References

1. J. Jumper *et al.*, Highly accurate protein structure prediction with AlphaFold. *Nature* **596**, 583-589 (2021).
2. M. Baek *et al.*, Accurate prediction of protein structures and interactions using a three-track neural network. *Science* **373**, 871-876 (2021).
3. N. Hiranuma et al., Improved protein structure refinement guided by deep learning based accuracy estimation. *Nature communications* **12**, 1340 (2021).
4. Z. Lin et al., Evolutionary-scale prediction of atomic-level protein structure with a language model. *Science* **379**, 1123-1130 (2023).
5. R. Wu *et al.*, High-resolution de novo structure prediction from primary sequence. *BioRxiv*, 2022.2007. 2021.500999 (2022).
6. A. Zemla, LGA: a method for finding 3D similarities in protein structures. *Nucleic acids research* **31**, 3370-3374 (2003).
7. D. A. Keedy *et al.*, The other 90% of the protein: Assessment beyond the Cαs for CASP8 template-based and high-accuracy models. *Proteins: Structure, Function, and Bioinformatics* **77**, 29-49 (2009).

8. Y. Zhang, J. Skolnick, Scoring function for automated assessment of protein structure template quality. *Proteins: Structure, Function, and Bioinformatics* **57**, 702–710 (2004).
9. V. Mariani, M. Biasini, A. Barbato, T. Schwede, lDDT: a local superposition-free score for comparing protein structures and models using distance difference tests. *Bioinformatics* **29**, 2722-2728 (2013).
10. J. Xu, Y. Zhang, How significant is a protein structure similarity with TM-score= 0.5? *Bioinformatics* **26**, 889-895 (2010).
11. L. N. Kinch, *et al.*, CASP9 target classification. *Proteins: Structure, Function, and Bioinformatics* **79**, 21–36 (2011).
12. G.N. Ramachandran, C. Ramakrishnan, V. Sasisekharan, Stereochemistry of polypeptide chain configurations. *Journal of Molecular Biology* **7**, 95–99 (1963).
13. V. B. Chen et al., MolProbity: all-atom structure validation for macromolecular crystallography. *Acta Crystallographica Section D: Biological Crystallography* **66**, 12-21 (2010).
